# Domain swapping: a mathematical model for quantitative assessment of structural effects

**DOI:** 10.1002/2211-5463.13911

**Published:** 2024-10-06

**Authors:** Irena Roterman, Katarzyna Stapor, Dawid Dułak, Leszek Konieczny

**Affiliations:** ^1^ Department of Bioinformatics and Telemedicine Jagiellonian University – Medical College Krakow Poland; ^2^ Department of Applied Informatics Silesian University of Technology Gliwice Poland; ^3^ ABB Business Services Sp. z o.o. ul Warszawa Poland; ^4^ Chair of Medical Biochemistry Jagiellonian University – Medical College Krakow Poland

**Keywords:** domain swapping, hydrophobicity, micelle‐like, protein structure

## Abstract

The domain‐swapping mechanism involves the exchange of structural elements within a secondary or supersecondary structure between two (or more) proteins. The present paper proposes to interpret the domain‐swapping mechanism using a model that assesses the structure of proteins (and complexes) based on building the structure of a common hydrophobic core in a micelle‐like arrangement (a central hydrophobic core with a polar shell in contact with polar water), which has a considerable impact on the stabilisation of the domain structure built by domain swapping. Domains with a hydrophobicity system that is incompatible with the micelle‐like structure have also been identified. This incompatibility is the form of structural codes related to biological function.

## Graphical abstract

The structure of a single chain interpreted on the basis of the hydrophobicity distribution (red line on profiles) represents the status with respect to an ideal hydrophobic core (blue) which is represented by a modified idealised hydrophobicity distribution (green line, upper part). Domain swapping constructs the hydrophobicity distribution in accordance with the idealised micelle‐like construction (lower part).

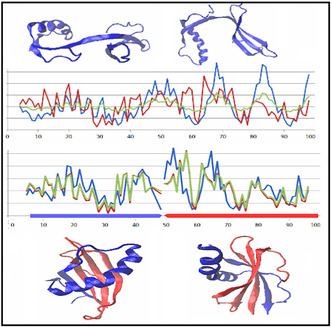

**Abbreviations**
3D Gauss: 3 dimensional form of Gauss function
3D: spatial, three dimensional
D_KL_
: Kullback–Leibler divergence entropy
*K*
: parameter modifying the 3D Gauss function
M: modified distribution of theoretical hydrophobicity
*O*
: distribution of observed hydrophobicity calculated as inter‐residual hydrophobic interaction
*O*/*R*
: relations between target distribution of hydrophobicity (*O*) in respect to R distribution –unified hydrophobicity distribution
*O*/*T*
: relations of observed (target distribution) versus theoretical (*T*) hydrophobicity distribution
PDB: Protein Data Bank
PDB – ID: Protein Data Bank Identification
R: unified hydrophobicity distribution
RD: relative distance
SARS‐CoV: severe acute respiratory syndrome coronavirus 2
*T*
: theoretical distribution of hydrophobicity accordant with 3D Gauss function spanned all over the protein molecule

The building mechanism for a domain consisting of fragments of two independent chains has been a subject of numerous studies [1, 2, 3, 4, 5, 6, 7]. The domain‐swapping mechanism is also associated with the phenomenon of misfolding, specifically in the context of multi‐chain fibril formation [8, 9]. In practice, the domain‐swapping phenomenon is used in the field of material design [10].

The present paper analyses this mechanism based on the structure of hydrophobic core. The assessment includes the contribution of these chain sections to the construction of a common hydrophobic core. The analysis aims to reveal the construction mechanism for a structure with a specific biological function, often also showing structuring that is far from a micelle‐like arrangement (lack of a centric hydrophobic core with a polar surface). Both proteins with a high degree of micelle‐like hydrophobicity ordering [11] and proteins with a relatively high degree of mismatch with the micelle‐like distribution have been identified [12]. Often, such a structure is obtained with the involvement of external factors, for example by chaperone proteins, such as chaperone [13], chaperonin [14] or prefoldin [15]. The mechanism of docking to the domain of ‘guest’ chain sections derived from distinct chains can be treated as common to ligand docking. This particularly applies to the docking of ligands involved in the construction of the hydrophobic core or locally to the hydrophobicity distribution in a given region of the target protein. Complexation of low‐molecular‐weight ligands, including ions in particular, is based on mainly electrostatic interactions with little influence on the type of hydrophobicity distribution.

The common feature between the binding of a ligand (including a substrate in particular) and a section of a polypeptide chain is the presence of a suitable cavity allowing the ligand to fit into the structure of this cavity. The assessment of the status of domains prepared to accept ‘guest’ type chain sections is carried out using the ‘fuzzy oil drop’ (FOD) model that takes into account the type of environment in which the protein is active (FOD‐M). This model determines the status of a given structural unit (in the present paper, the status of domains) by means of the values of two parameters: RD (relative distance) expressing the degree to which the hydrophobicity distribution present in a given protein is consistent with an idealised distribution matching a micelle‐like distribution (centrally located hydrophobic core with a polar surface); and *K* expressing the degree to which an environment different from polar water causes a blurring of the hydrophobic core. The assessment is concerned with determining the status of a domain with a ‘guest’ chain fragment and the status without this fragment. This is to determine the contribution of the ‘guest’ fragment to the construction of the common hydrophobic core or to oppose such a contribution.

The proteins discussed here are associated with the activity of the SARS‐CoV main protease, whose structure is available in Protein Data Bank (PDB) [16] in many different forms (single domains, chains, complexes) [17, 18, 19, 20]. In order not to be limited to one specific example, a different protein with a different biological activity was referenced—human stefin B [21].

## Materials and methods

### Data

The analysis concerns proteins representing the SARS‐CoV‐2 main protease and cystatin B as a reference protein. A summary of the proteins analysed is given in Tables 1 and 2. Presenting the list of human cystatin mutants.

**Table 1:** Summary of proteins analysed.

PDB – ID	Biological activity	Source organism	Ref
2K7X	c‐Terminal domain of SARS‐CoV main protease	*Sars coronavirus*	[17]
2H2Z	SARS‐CoV main protease with authentic N‐ and C‐termini	*Sars coronavirus*	[18]
1UK2	SARS‐CoV main proteinase (3clpro) at ph8.0	*Sars coronavirus*.	[19]
3EBN (AO‐M (pro))	Dimerisation of SARS‐CoV main protease C‐terminal domain due to domain swapping	*Sars coronavirus*	[17]
3IWM (M (pro))	Octameric SARS‐CoV main protease	*Sars coronavirus*	[20]
**Reference proteins**			
2OCT	Cystatin B	*Homo sapiens*	[21]
1N1C	Chaperone	*Shewanella massilia*	[22]
3VM9	Myoglobin	*Equus caballus*	[23]
1JWI	Toxin	*Bitis arietans*	[24]
3NBS	Cytochrome	*Equus caballus*	[25]
3S1U	Transferase	*Thermoplasma acidophilum*	[26]
4AZM	Lipid binding	*Homo sapiens*	[27]
4P2X	Lyase	*Mycobacterium tuberculosis*	[28]
4ZCB	Lipid binding	*Homo sapiens*	[29]
5U6G	Transport	*Homo sapiens*	[30]
6J52	Apoptosis	*Frog virus*	[31]
6YRE	Transferase	*Thermoplasma acidophilum*	[32]
11BA	Hydrolase	*Bos taurus*	[33]
1JS0	Hydrolase	*Bos taurus*	[34]
1TIJ	Inhibitor	*Homo sapiens*	[35]
4ESK	Immune system	*Mus musculus*	[36]
6QKY	Lyase	*Streptococcus pneumoniae*	[37]
6TP9	Electron transport	*Pseudomonas aeruginosa*	[38]

**Table 2:** List of analysed mutants of human cystatin c.

Structural effects of mutations in human cystatin B			
PDB – ID	Mutation	Source organism	Ref
3QRD	L68V	*Homo sapiens*	[39]
3S67	V57P	*Homo sapiens*	[40]
3SVA	V57D	*Homo sapiens*	[40]
3PS8	L68V	*Homo sapiens*	[40]

### Model description

A description of the model is available in other papers [11, 12, 13]. For the purposes of the present paper, the description is reduced to a minimum to avoid redundancy.

It is based on the assumption that a polypeptide chain folding in an aqueous environment adopts a structural form as close as possible to that of a micelle with a centrally located hydrophobic core and a polar surface. The mathematical representation of this type of hydrophobicity distribution is a 3D Gaussian function. This function, spanned over the protein body, encapsulates the given protein by adjusting the parameters of this function (σ_*X*_, σ_*Y*_ and σ_*Z*_) to the size and shape of the protein discussed. The value of the 3D Gaussian function in the position of the effective atoms (the averaged position of the atoms comprising the given amino acid) expresses the level of hydrophobicity expected for idealised structuring. This level is referred to by the value *T*_*i*_ (theoretical hydrophobicity level).

The *T* distribution is expressed by the function:

(1) $$H_{i}^{T}=\frac{1}{H_{\mathrm{sum}}^{T}}\exp\left(\frac{-{\left(x_{i}-\bar{x}\right)}^{2}}{2\sigma_{x}^{2}}\right)\exp\left(\frac{-{\left(y_{i}-\bar{y}\right)}^{2}}{2\sigma_{y}^{2}}\right)\exp\left(\frac{-{\left(z_{i}-\bar{z}\right)}^{2}}{2\sigma_{z}^{2}}\right)$$

The level of idealised hydrophobicity will be called *T*_*i*_ later in this paper. The expression $1/H_{\mathrm{sum}}^{T}$ is the normalisation factor. The *H* ^*T*^ with the index sum denotes the summary of all *H*_*i*_ calculated for *T* distribution.

The hydrophobicity distribution in the actual protein does not necessarily achieve an ordering consistent with the idealised one. The actual hydrophobicity level is an expression of the hydrophobic interaction depending on the distance between the effective atoms and the intrinsic hydrophobicity of the interacting amino acids. The *O*_*i*_ (observed) record was used to express this type of level. A function record expressing this type of interactions proposed in [41] was adopted.

(2) $$H_{i}^{O}=\frac{1}{H_{\mathrm{sum}}^{O}}\sum_{j}\{\begin{array}{c} \left(H_{i}^{r}+H_{j}^{r}\right)\left(1-\frac{1}{2}\left(7{\left(\frac{r_{\mathrm{ij}}}{c}\right)}^{2}-9{\left(\frac{r_{\mathrm{ij}}}{c}\right)}^{4}+5{\left(\frac{r_{\mathrm{ij}}}{c}\right)}^{6}-{\left(\frac{r_{\mathrm{ij}}}{c}\right)}^{8}\right)\right)\;\text{for}\;r_{\mathrm{ij}}\leq c\\ 0,\\ \;\text{for}\;r_{\mathrm{ij}}>c \end{array}$$

The $1/H_{\mathrm{sum}}^{O}$ is the normalisation factor. The *H* ^*O*^ with the index sum denotes the summary of all *H*_*i*_ calculated for *O* distribution.

The comparison of the *T* and *O* distributions is possible to a quantitative degree by using divergence entropy introduced by Kullback–Leibler [42].

(3) $$D_{\mathrm{KL}}\left(P|Q\right)=\sum_{i=1}^{N}P_{i}\log_{2}\frac{P_{i}}{Q_{i}}$$

where *P*—distribution under consideration (the *O* distribution in our analysis) and *Q*—reference distribution (*T* distribution in our analysis).

Using the notation applied in this paper the Eqn (3) takes the form:

(4) $$D_{\mathrm{KL}}\left(O|T\right)=\sum_{i=1}^{N}O_{i}\log_{2}\frac{O_{i}}{T_{i}}$$

The *D*_*KL*_(*O*|*T*) designation was adopted, expressing the ‘distance’ between the *O* distribution (studied distribution) and the *T* distribution (reference distribution). However, the value determined this way cannot be interpreted. Therefore, another reference distribution *R* is introduced where all the values *R*_*i*_ *= 1*/*N*, where *N* is the number of amino acids in the chain. Such a distribution represents a state of uniform hydrophobicity distribution within the protein and therefore devoid of a hydrophobic core. The determined *D*_*KL*_(*O*|*R*) value defines the ‘distance’ between the observed distribution and the distribution devoid of any variation in hydrophobicity levels.

By comparing the values of *D*_*KL*_(*O|T*) and *D*_*KL*_(*O|R*), it is possible to identify a distribution to which the observed distribution is similar. In order to express the ‘similarity’ of the *O* distribution to one of the two reference distributions, the RD parameter was introduced, which defines the RD calculated as the value of the quotient numerator: *D*_*KL*_(*O|T*), denominator: *D*_*KL*_(*O|T*) + *D*_*KL*_(*O|R*). Thus determined, RD < 0.5 indicates the presence of a hydrophobic core, while RD > 0.5 indicates the absence of a hydrophobic core.

(5) $$\mathrm{RD}=\frac{D_{\mathrm{KL}}\left(O|T\right)}{D_{\mathrm{KL}}\left(O|T\right)+D_{\mathrm{KL}}\left(O|R\right)}$$

An RD value < 0.5 describing a given protein indicates the presence of a hydrophobic core, which means that the folding process took place according to a micelle‐like scenario. Therefore, the RD value determines the degree of reconstitution of the micelle‐like arrangement. The dissimilarity of distribution is based on a local excess of hydrophobicity—this implies a protein surface prepared to interact with another protein with similar exposure [43]. A local deficiency in hydrophobicity (*O*_*i*_ *< T*_*i*_) often indicates the presence of a ligand (substrate) binding cavity [12]. The status of the protein after elimination of amino acids showing significant dissimilarity results in a reduction of the RD value, leading to the identification of a part of the protein with RD < 0.5, which indicates its purpose as responsible for the solubility of the given protein.

However, the aqueous environment is not the only one in which proteins show their activity. Such a different environment is the cell membrane, where, to stabilise the protein in the membrane environment, hydrophobic residues are expected to be exposed on the surface, while polar amino acids (mainly from the membrane channel) are located in the centre. This distribution of hydrophobicity can be reproduced using a function ‘complement’ to the 3D Gaussian function, in the form: *T*_MAX_ – *T*_*i*_ where *T*_MAX_ is the maximum value for the *T* distribution in the analysed protein. In the light of previous studies, the *O* distribution of membrane proteins turns out to match the modified *M*_*i*_ distribution in the form of

(6) $$M_{i}={\left[T_{i}+K\times {\left[T_{\mathrm{MAX}}-T_{i}\right]}_{n}\right]}_{n}$$

where the *n* index denotes the normalisation of the distribution. The determined value of the *K* parameter for a given protein is interpreted as the degree of contribution of the environment different from the polar environment.

The identified status of a protein with a low RD value and *K* = 0.0 is interpreted as a protein reproducing a micelle‐like arrangement in the polar (aqueous) environment. Such proteins have been identified: down‐hill, fast‐folding, ultra‐fast‐folding and antifreeze type II proteins [11]. The identification of these proteins confirms the validity of the model adopted.

The membrane proteins show RD values > 0.5 and *K* values > 1.0 [12]. It appears that, in many cases, water‐soluble proteins show a micelle‐like distribution of hydrophobicity [44, 45]. These include many enzymes that create a specific environment for a specific catalytic reaction by providing the external force field for the enzymatic cavity [46].

Proteins folded with chaperone proteins (prefoldin [15], chaperone [13] or chaperonins [14]) show a micelle‐like distribution by adapting, during folding, to the environment imposed by the chaperone protein.

Identifying the presence of cavity (local hydrophobicity deficiency) may prove useful for drug design by indicating the site of potential ligand binding.

**Fig. 1:**
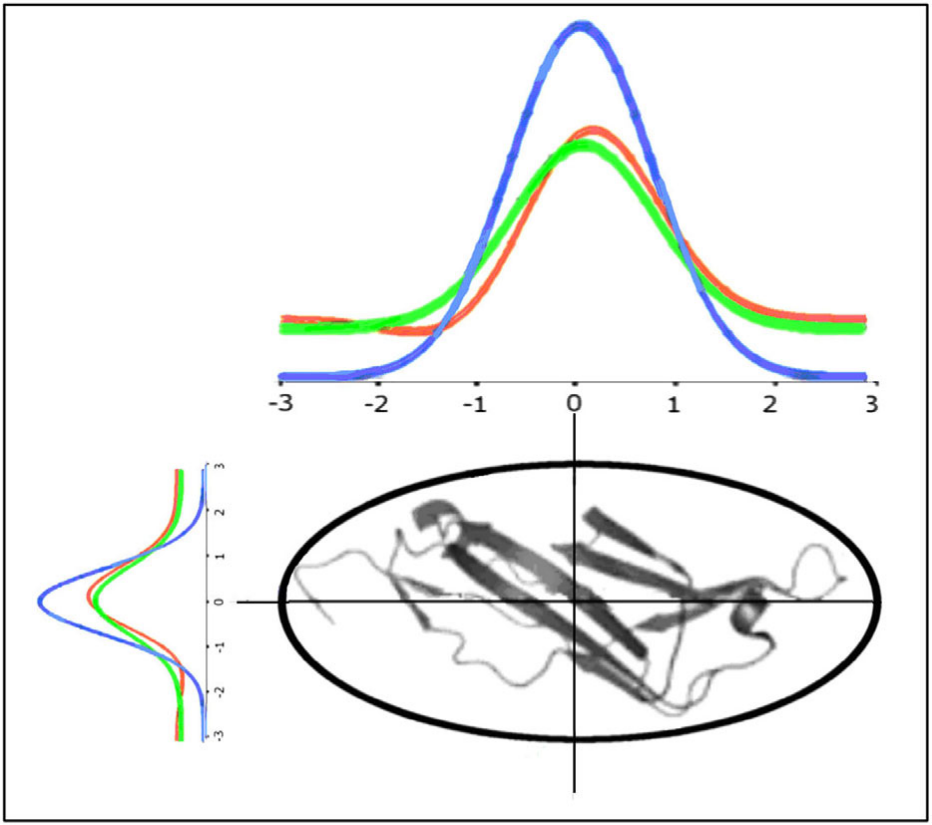
The protein under consideration encapsulated in 3D Gauss function (here expressed in 2D presentation) may be represented by idealised distribution (micelle‐like)—blue line. The real hydrophobicity distribution (effect of inter‐residual hydrophobic interaction—red line) differs in respect to idealised one. The green line represents the *M* function which expresses the external force field in which the protein was folded.

The graphic presentation of the applied model is shown in Fig. 1. The protein encapsulated in 3D Gauss function can be represented by the *T* function (idealised hydrophobicity distribution—blue line—Eqn 1). The observed hydrophobicity distribution (*O* distribution—result of inter‐residual interaction—Eqn 2) represented by red line differs versus the idealised. The search for M distribution (green line—Eqn 6) representing the best fit of *T* function to *O* distribution allows the prediction of conditions in which the folded protein followed the external force field, which differs in respect to polar water.

### Experimental research

The topic of the current work is *in silico* analysis. Experimental research on the phenomenon in question is conducted in laboratories, where a monomer to dimer transition mechanism is suggested [45]. In these works, attention is paid to the role of hydrogen bonds in the discussed transformation [46, 47].

The phenomenon of the observed multi‐chain structure based on domain swapping is also associated with the misfolding process [3, 12, 48, 49].

It is also assumed that domain swapping obtained through biomedical engineering experiments can influence biological activity in a controlled manner [39].

The model presented here draws attention to the factor of hydrophobic interaction, which, in addition to disulfide bonds, is treated as an important factor in stabilising the tertiary structure. Therefore, this factor can be treated as important in the process of generating a structure stabilised by the presence of a hydrophobic core common to the two polypeptide chains.

## Results

The procedure used in the present analysis consists of the following steps:

1. A 3D Gaussian function is spanned over the structural unit (domain), which includes fragments from different chains.
2. The hydrophobicity distribution based on the 3D Gaussian function is determined, indicating the level of theoretical (idealised) hydrophobicity expressed as *T*_*i*_ in the positions of effective atoms (the averaged position of atoms comprising a given amino acid).
3. The observed distribution—the actual distribution resulting from the interaction of the inter‐residual hydrophobicity—is determined by assigning the *O*_*i*_ values to the successive positions of the effective atoms.
4. Both distributions are normalised.
5. The magnitude of the differences between the *T*_*i*_ and *O*_*i*_ distributions is determined by means of *D*_*KL*_ divergence entropy for the *O*_*i*_ *|T*_*i*_ relationship.
6. In order to interpret the status of a given structural unit, the *R* distribution with uniform hydrophobicity levels is determined—this distribution represents the situation of uniform distribution of hydrophobicity within a given protein.
7. The determined value of *D*_*KL*_ divergence entropy for the *O*_*i*_ *|R*_*i*_ relationship defines the ‘distance’ of the *O*_*i*_ distribution towards a distribution without the hydrophobic core.
8. To assess the proximity of the *O*_*i*_ distribution to one of the mentioned, the RD value is determined.
9. In the next step, the value of the *K* parameter is determined, which defines the degree of contribution of factors altering the specificity of the aqueous environment (reduced polarity of the protein environment).
10. All the operations mentioned in items 1–9 are conducted for a structural unit without a chain fragment derived from another protein molecule.

In the light of this procedure, all the proteins in question are characterised using the RD and *K* parameter values for both the status of the domain containing the ‘guest’ chain and the part of the domain without the ‘guest’ chain fragment.

The summary results of the analysis of the proteins mentioned (Table 2) are given in Table 3. It includes a description of the status of SARS‐CoV main protease and a description of a reference protein with a different biological role (cystatin B from *Homo sapiens*—PDB – ID – 2OCT).

**Table 3:** Summary of results in the form of RD and *K* parameter values characterising the discussed domains formed or involved in domain swapping. D symbol indicates the domain. D1 identifies the domain in the chain—numbered consecutively, starting from the N‐terminal. DA denotes a domain derived from chain A. DS denotes a swapping domain.

PDB – ID	Structural unit	Chain fragment	RD	*K*
2K7X	Domain	(187–306)	0.504	0.4
2H2Z	Monomer		0.671	1.0
	D1	(1–14) (100–197)	0.417	0.1
	D2	(15–99)	0.407	0.2
	D3	(198–301)	0.379	0.2
1UK2	Dimer		0.713	1.0
	Chain A		0.655	0.9
	AD1	(3–14) (100–197)	0.369	0.2
	AD2	(15–99)	0.425	0.3
	AD3	(198–301)	0.375	0.2
3EBN	Complex ABCD – D3	4 × D3 198–297	0.807	2.2
	Chains A + C		0.827	2.3
	Chain A	198–297	0.736	1.0
	Chain C		0.741	1.1
	DS AC	A (198–221) + C (224–298)	0.408	0.3
	DS CA	C (198–221) + A (224–298)	0.396	0.3
3IWM	Complex	A, B, C, D	0.827	2.3
	Chain A	A	0.704	1.0
	Chain A, C	A, C	0.827	2.3
	DS AC	A (197–221) + C (224–300)	0.366	0.2
	DS CA	C (197–221) + A (224–300)	0.391	0.2
	A‐D1	(1–98)	0.431	0.2
	A‐D2	(99–192)	0.351	0.2
	A‐D3	(193–301)	0.656	0.8
	A‐D12	(1–192)	0.414	0.3
**Reference protein**				
2OCT	Complex AB		0.643	1.0
	Chain A		0.595	0.7
	Chain B		0.625	0.8
	Domain	A (2–49) + B (47–98)	0.536	0.5
	Domain	B (5–49) + A (49–98)	0.469	0.1

### C‐terminal domain of SARS‐CoV main protease (PDB – ID – 2K7X)

**Fig. 2:**
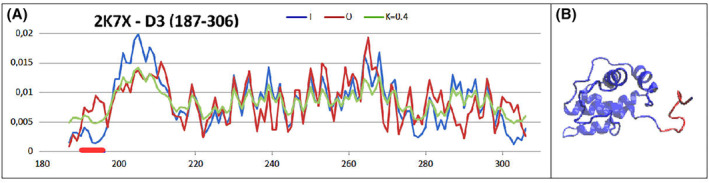
Dom3 analysis of SARS‐CoV main protease. (A) Set of *T*, *O* and *M* profiles for the value *K* = 0.4. (B) 3D structure with a highlighted loose loop (red), the removal of which results in a lower RD value < 0.5—this red section is highlighted on the *x*‐axis (A).

The status of a single domain represented by a protein present in the PDB database with the identifier 2K7X, representing the 187–306 section shows a status very similar to the micelle‐like form, minimally exceeding the cut‐off value for RD = 0.5. Elimination of a loose fragment of the N‐terminal loop (191–195—marked in Fig. 2A,B as the red line on the *x*‐axis) results in a considerable reduction of the RD value to 0.436. The very low value of the *K* parameter indicates structuring achieved mainly by the involvement of the polar water environment. The contribution of domain swapping is not present in this structure. In conclusion, it should be stated that this domain shows a micelle‐like ordering of hydrophobicity.

### Monomer structure of SARS‐CoV main protease (PDB – ID – 2H2Z)

**Fig. 3:**
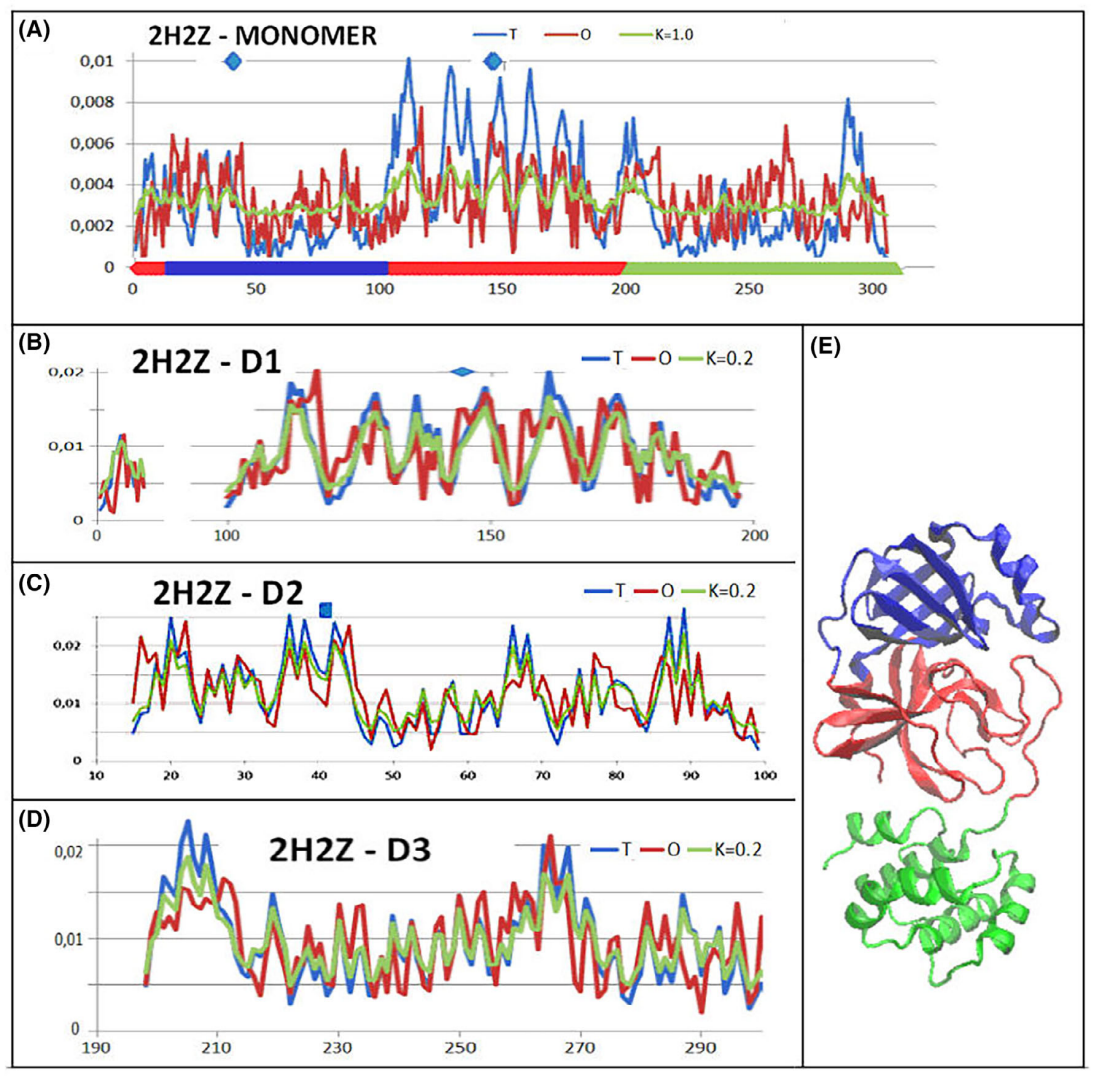
Set of *T*, *O* and *M* profiles for respective values of the *K* parameter (given in the legends to the profiles). (A) Complete chain—upper line—catalytic residues—blue dots. (B) Domain D1—red fragment on A—blue dot on top line—catalytic residue. (C) Domain D2—blue fragment on A—blue dot on top line—catalytic residue. (D) Domain D3—green fragment on A. (E) 3D presentation with domains distinguished by colours as on *x*‐axis shown in A.

The example of chain A of main protease SARS‐CoV‐2 (PDB – ID – 2H2Z) reveals how a chain structure with high values of the RD and *K* parameters can be constructed from the components: domains 1, 2 and 3 (as shown in Fig. 3B–D). The domain status shows an extremely high match between the hydrophobicity arrangement with the micelle‐like arrangement. In contrast, the complete chain in the arrangement of linearly positioned domains results in an arrangement with a significant degree of mismatch with the micelle‐like arrangement. The summary of the *T*, *O* and *M* profiles for a complete chain (Fig. 3A) reveals a relative match between *T* and *O* distributions for the N‐ and C‐terminal domains Dom 2 (blue) and Dom3 (green). However, the domain constructed from the central chain fragment (D1—distinguished in Fig. 3A—red line on *x*‐axis) shows a significant deficiency in hydrophobicity.

The positions of the catalytic residues in relation to the domain structures demonstrate an almost perfect match of hydrophobicity levels. However, their status within the structure of a complete chain shows a significant divergence the *O*_*i*_ and the *T*_*i*_ level, which gives these residues a specific status. These residues are additionally placed in the external force field (for the catalytic centre, the external force field is provided their ordering within the complete protein molecule) [44].

### SARS‐CoV coronavirus main proteinase (3clpro) at ph8.0 (PDB – ID – 1UK2)

The next step in the construction of the SARS‐CoV main protease combination is a dimer composed of the complete A and B chains.

**Fig. 4:**
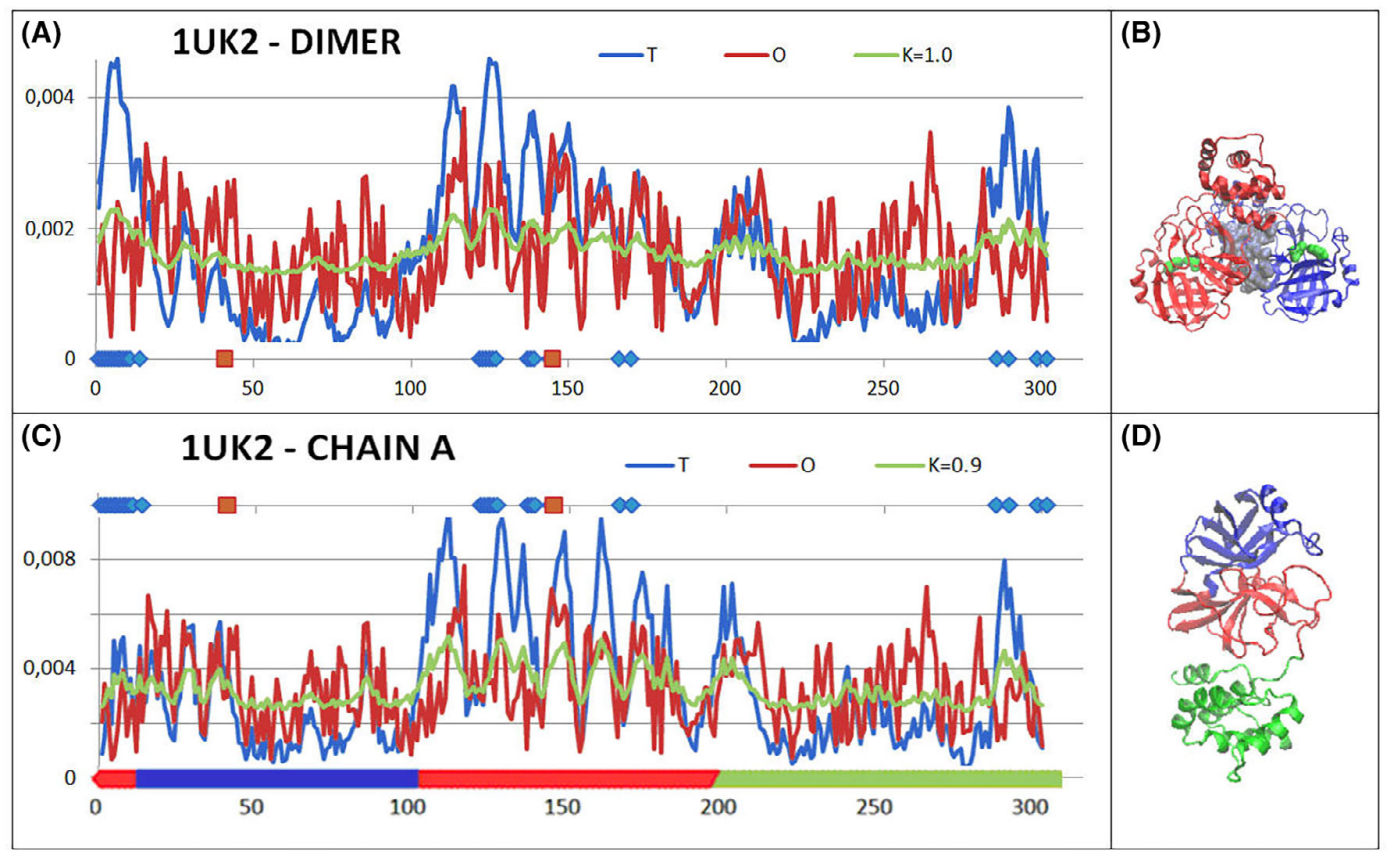
Set of *T*, *O* and *M* profiles for the corresponding value of the *K* parameter (given in the legend). (A) Dimer—profiles for the two chains applied according to numbering. The *x*‐axis shows positions involved in interactions with the second chain (blue dots) and red dots—positions of catalytic residues. (B) 3D presentation of the dimer structure. Chain A—red, Chain B—blue, catalytic residues—green, residues distinguished as ice‐blue (space filling)—interchain interface. (C) *T*, *O* and *M* profiles for a single chain. Residues involved in an interaction with the second chain—blue dots on top line, catalytic residues—red dots on top line. (D) 3D presentation of a single chain with highlighted domains as shown in colour in B—bottom line.

The dimer status in terms of the FOD‐M model shows considerable deviations from the micelle‐like distribution (Fig. 4A). The *M* distribution for a high *K* value assumes a form similar to a straight line (*R* distribution). Dom1 with few residues from Dom3 is predominantly involved in the construction of the interface.

The status of a single chain is similar to that represented in the 2H2Z structure (Fig. 3). The status of the catalytic residues is determined by a local excess of hydrophobicity for the 41H and a local deficiency of the 145C residue. Within the chain treated as an individual structural unit, the 41H residue appears to represent a status consistent with that expected by matching the local micelle‐like ordering, while the 145C residue is located in close proximity to the cavity (the environment shows a significant hydrophobicity deficiency).

**Fig. 5:**
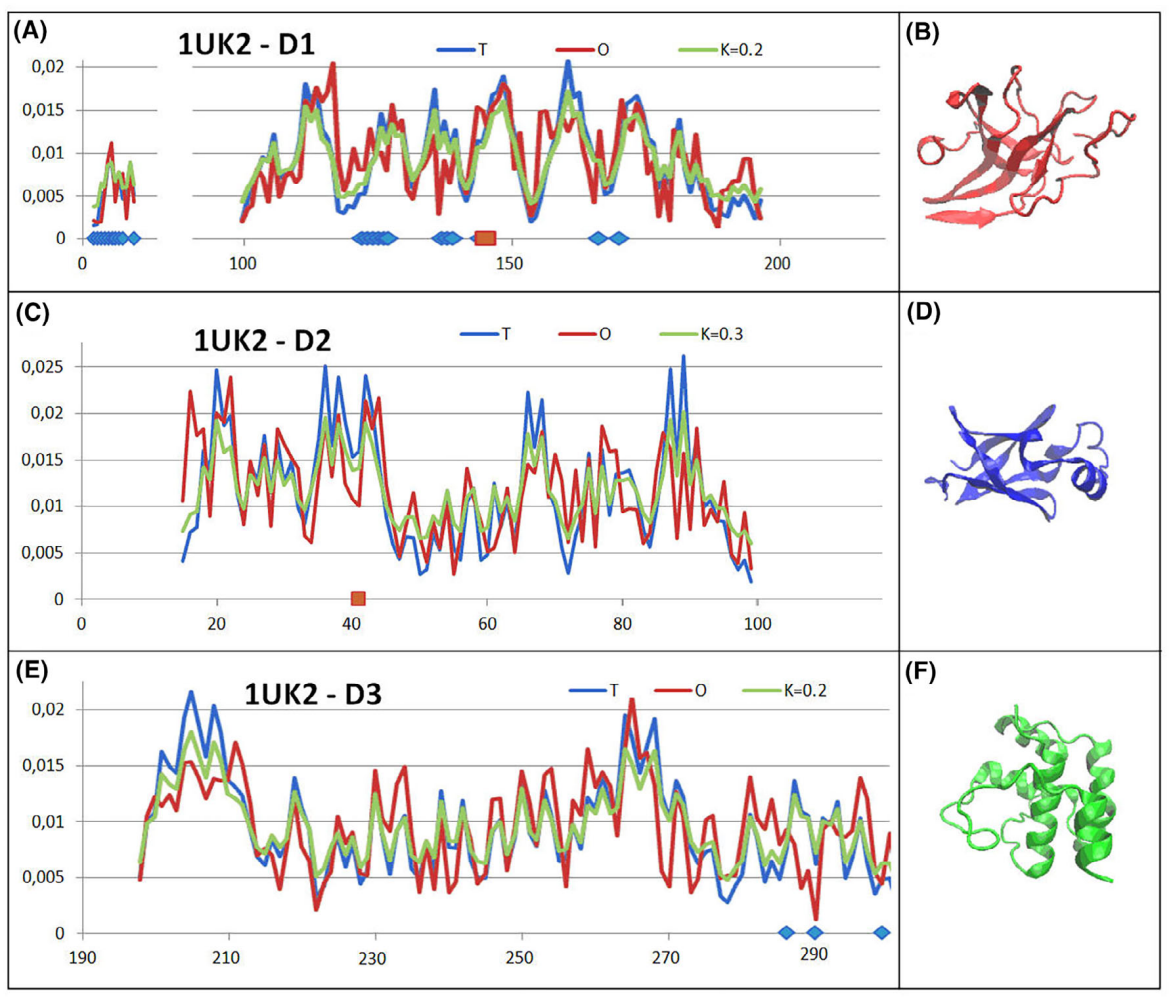
Set of profiles for *T*, *O* and *M* distributions as part of domains treated as individual structural units (a 3D Gaussian function generated for a domain) as observed in the structure PD ID—1UK2. (A, C, E) Set of profiles for subsequent domains. Positions of catalytic residues—red dots on bottom lines, blue dots—residues engaged in an interchain interaction. (B, D, F) 3D presentations of individual domains—colours as shown in Fig. 4C.

The status of the domains as part of a single chain (Fig. 5) appears to be very similar to that represented in 2H2Z (Fig. 3).

The summary of the *T*, *O* and *M* profiles for domains treated as individual structural units (a 3D Gaussian function generated for each domain independently) indicates very high match between the *O* distributions and the *T* distributions. The *M* distributions very similar to the *T* distributions for each domain (Fig. 5). The status of the catalytic residues within the domains turns out to be very consistent with the one expected according to the idealised distribution.

### Special dimerisation of SARS‐CoV main protease (PDB – ID – 3EBN)

The domain‐swapping structure is present in the structure of the complex consisting of four D3 domains with respect to the A (198–221) + C (224–298) and A (224–298) + C (198–221) sections. A summary of the RD and *K* parameter values describing this complex given in Table 3 identifies the complex in both tetramer and dimer forms as representing a high disorder of the hydrophobicity distribution. The high values of the *K* parameter (*K* > 2.0) suggest a significant environmental contribution to the stabilisation of the observed structure.

The structure of single chains—the D3 domains—represents a status with lower RD and *K* values with a distribution exceeding the threshold for RD = 0.5.

Domains obtained by means of domain swapping show low RD and *K* values, which is interpreted as an adaptation to the structure preferred by the polar water environment with a centrally located hydrophobic core.

### Dimer structure of octameric SARS‐CoV main protease (PDB – ID – 3IWM)

**Fig. 6:**
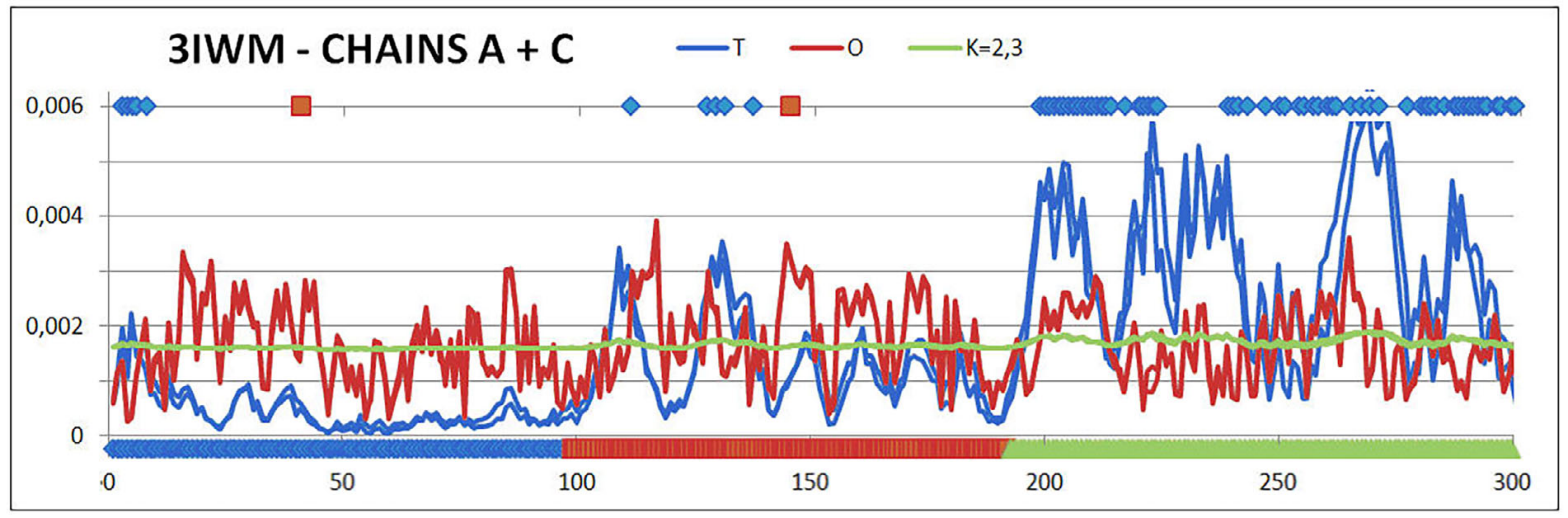
Set of profiles *T*, *O* and *M* for the dimer (chains A and C) as observed in the PDB structure ID 3IWM. On the bottom axis, the chain fragments corresponding to the consecutive domains are marked in colour. Blue dots—top line—residues engaged in interchain interaction. Red dots top line—positions of catalytic residues.

The status of a four‐chain complex is far from the micelle‐like arrangement, showing the value *K* = 2.3 (Table 3, Fig. 6). This observation indicates a significant contribution of the modified environment in the generation of the structure or the presence of a ‘permanent chaperone’ in the form of, for example, a second chain in the complex.

**Fig. 7:**
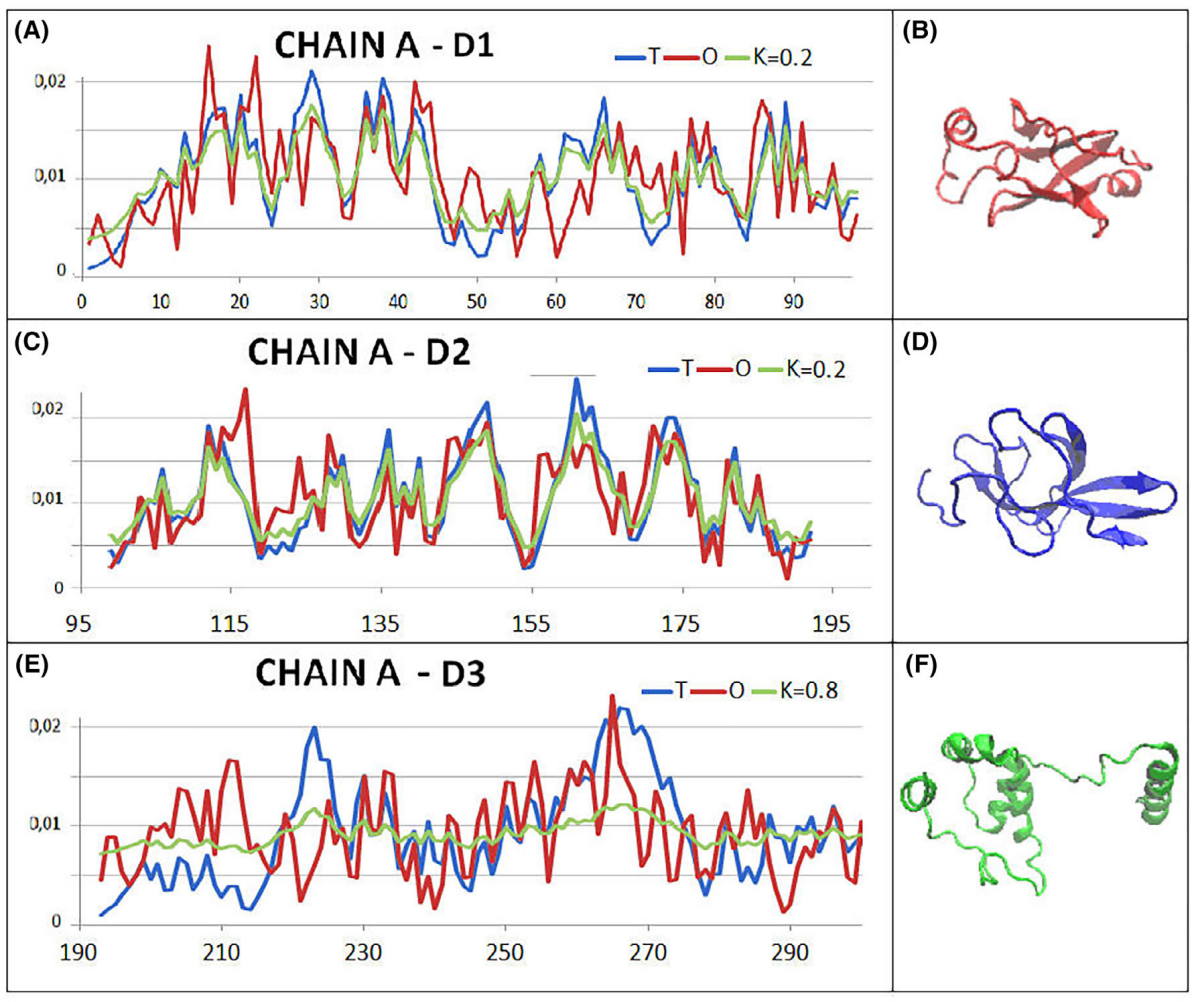
Summary of the *T*, *O* and *M* profiles (for respective values of the *K* parameter given in the legend to the respective figure)—3IWM. (A) Dom1—the set of profiles reveals a hydrophobicity distribution matching the micelle‐like arrangement. (B) 3D presentation of Dom1. (C) Dom2—the set of profiles *T*, *O* and *M* indicates an ordering of hydrophobicity distributions matching the micelle‐like arrangement. (D) 3D presentation of Dom2, where a globular form is visible (similar to Dom1). (E) Dom3—the set of profiles *T*, *O* and *M* reveals a rather significant divergence between the *O* distribution and the *T* distribution. The high value of the parameter *K* = 0.8 suggests an arrangement far from the micelle‐like arrangement. (F) 3D presentation of Dom3 revealing a form significantly different from a globular structure as a helix clearly mismatched with the domain arrangement (right site). Red dots on A and C on bottom line—position of catalytic residues.

The diverse status of the domains is visible by comparing the *T*, *O* and M profiles for the appropriate values of the *K* parameter (Fig. 7). Dom1 and Dom2 show a micelle‐like order with very low parameter values. Dom3, on the other hand, represents a status that excludes the presence of a hydrophobic core, which is formed only after the incorporation of a chain fragment in the domain‐swapping system.

**Fig. 8:**
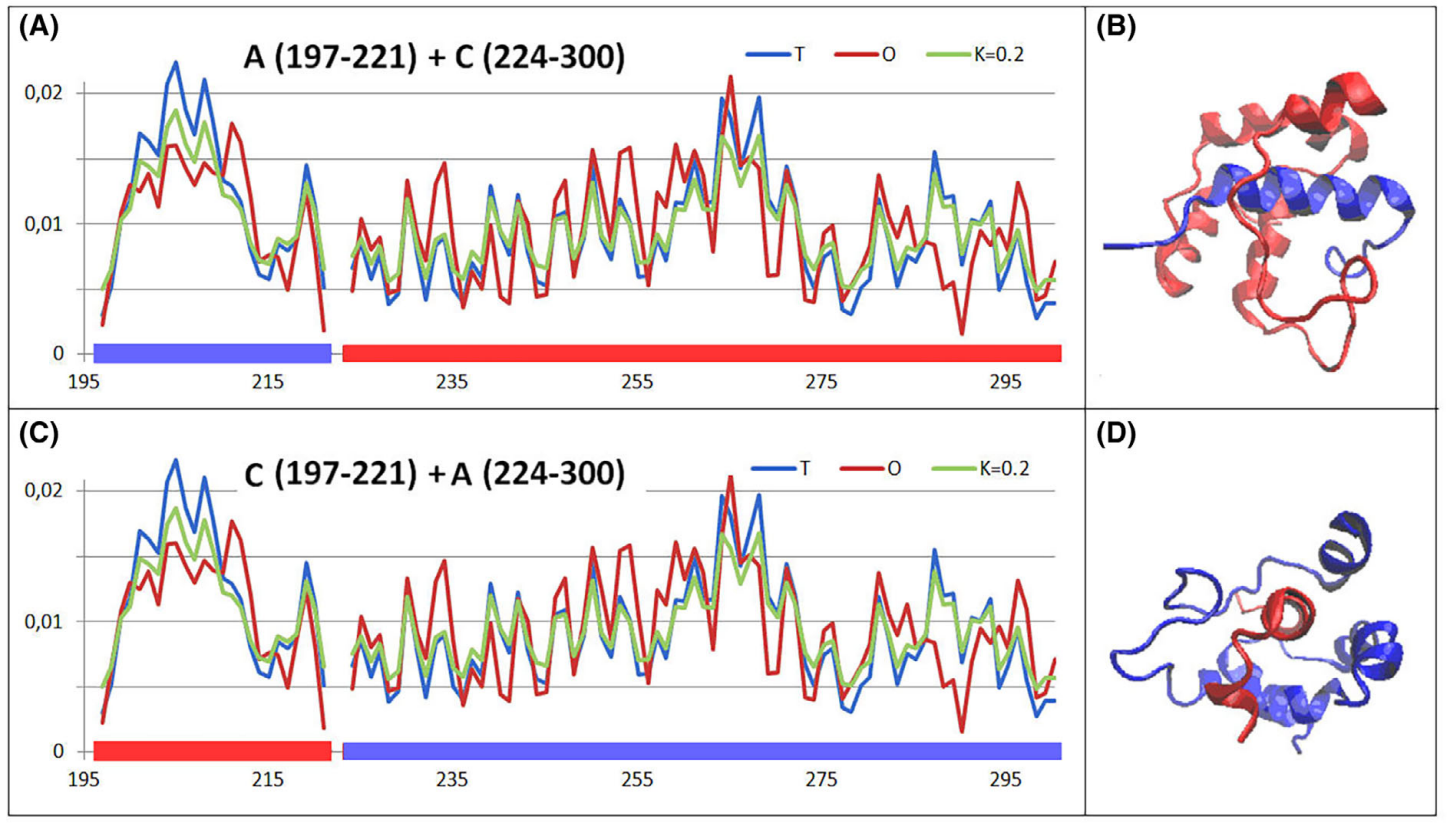
Set of the *T*, *O* and *M* profiles for domains whose structure is obtained by domain swapping. (A) A summary of chain A fragments remaining in the complex with chain C section (numbering given in the legend to the profiles). (B) 3D presentation of domain swapping consisting of the chain sections shown in A. Colours as shown in A. (C) A summary of chain A fragments remaining in the complex with chain C section (numbering given in the legend to the profiles). (D) 3D presentation of domain swapping consisting of the chain sections shown on A. Colours as shown in A.

The situation is clarified in the domain‐swapping analysis, where the insertion of a helical section causing a significant deviation from the globular arrangement into the second chain domain results in an ordering highly matching the micelle‐like arrangement (Fig. 8).

In a summary of the SARS‐CoV main protease dimer structure analysis, it should be concluded that the domain‐swapping mechanism results in a locally stable structure of the arrangement, which, considering the dimer form, shows a status deviating from the micelle‐like arrangement. Such an arrangement is not supported by an external force field such as the polar water environment. It is, however, obtained by means of an arrangement of domains that all individually exhibit a status of micelle‐like ordering. Some of them acquire such a status when a section derived from the second polypeptide chain is implemented.

The analysis of all the structural forms discussed above shows highly intentional structuring of all the components to obtain a stable structural form exhibiting enzymatic activity using structuring deviating from micelle‐like structuring. As shown in Ref. [44], in the case of enzymes, protein body is the provider of the appropriate external force field for the catalytic centre. The specificity of this field is probably closely related to the process catalysed by the enzyme, hence the high variation in the forms of this external force field. As shown in the example of the protein discussed herein, one way to obtain a stable form of structuring different from the one preferred by the aqueous environment (micelle‐like) is through a mechanism based on domain swapping. A form of dynamic structural changes accompanying the activity is probably also encoded in the construction system of chain section swapping leading to a stable form. In the case of domain swapping, one can speculate about the stability of these domains.

### Reference proteins

#### Human cystatin B (PDB – ID – 2OCT)

**Fig. 9:**
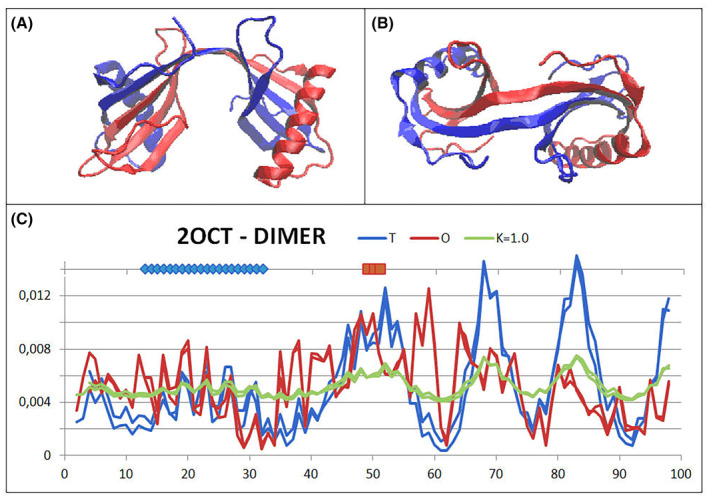
3D structure of the cystatin B dimer. (A, B) Two different orientations of the chain arrangement—chains with different colours. (C) The set of *T*, *O* and *M* profiles for the dimer blue dots on top line—helical fragment involved in ‘swapping’. Highlighted red fragment on top line—a section linking two domains (in A—a section in the form of an interdomain linker in the central part of the presentation).

The reference protein—cystatin B from *H. sapiens*, belongs to the cysteine proteinase inhibitor group (PDB – ID—2OCT). It represents specific structure where the swapping of chain sections appears to be important for dimer stability. The ‘guest’ section covers almost the entire length of the dimer molecule contributing an important element of stabilisation, especially within the linker of the two distinct domains (Fig. 9). A single chain that cannot generate a Beta structure on its own would not be able to maintain the rigidity of two independent domains.

The status of the complete dimer arrangement shows a significant mismatch with the micelle‐like arrangement. The high *K* value indicates the need for an environmental factor to stabilise this form of the arrangement.

Single chains are also clearly not matched with the micelle‐like arrangement. In contrast, the status of domains obtained by domain swapping appears to be clearly matching an arrangement with a centric hydrophobic core and a polar shell. This implies a high solubility of the domains and, consequently, of the entire dimer. For the complete domain with composition A (2–49) and B (47–98), this status is determined by an RD value above the cut‐off level (threshold) RD = 0.5. In contrast, by eliminating the 3 N‐terminal residues and defining the swapping section of the B chain (49–98), the status of this arrangement turns out to be very highly matching the micelle‐like arrangement. The very low value of the *K* parameter indicates a high match with the arrangement preferred by the polar water environment.

The set of *T*, *O* and *M* profiles for the dimer reveals a rather high consistency of the *O*_*i*_ and *T*_*i*_ levels for the helical sections. It can be inferred that they have a significant contribution to the stabilisation of the dimer as a whole. The linker section also seems to play a similar role in stabilisation. In contrast, the β‐sheet sections show a significant local deficiency in hydrophobicity.

**Fig. 10:**
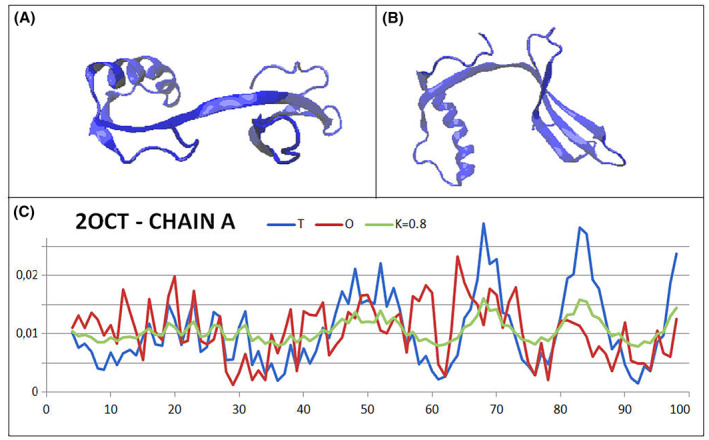
The structure of a single cystatin B chain. (A, B) Two different perspectives of the 3D presentation of the single chain structure. (C) The set of *T*, *O* and *M* profiles revealing a significant mismatch with the micelle‐like arrangement.

Figure 10 reveals a mismatch between the chain structuring and the arrangement preferred by the polar water environment. Such an arrangement is possible with the participation of a ‘permanent chaperone’ in the form of the second chain, whose contribution to the construction of stable domains results in the maintenance of a form far from the globular arrangement.

**Fig. 11:**
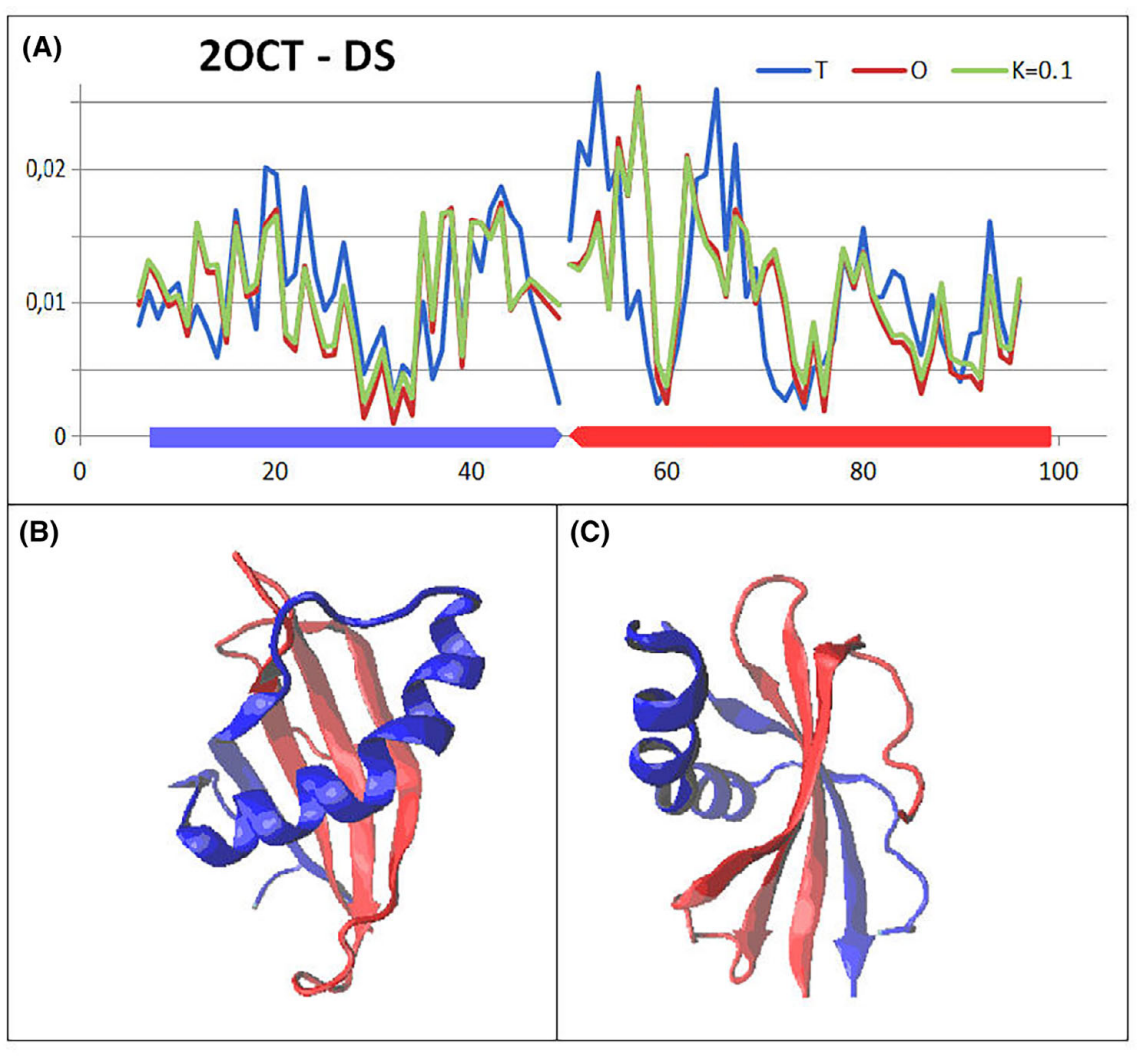
Domain containing a ‘guest’ chain fragment—chain A (2–49)—blue fragment with chain B (49–98)—blue. (A) The *T*, *O* and *M* profiles (for *K* = 0.1) identified for a domain composed of fragments of two chains—sections originating from chain A and B are colour‐coded on the horizontal axis. (B, C) Two 3D orientations of the structure of the domain made up of sections—as shown in A.

The stabilisation of domains built by means of the domain‐swapping mechanism is visualised in Fig. 11, where the domains achieve significant stabilisation through the swapping of chain sections.

Simulation in an environment expressed with higher values of the *K* parameter representing an altered environment is planned to verify the assumed amyloid transformation model [12]. The present paper, however, concentrates on the phenomenon of domain swapping as a means of obtaining structures that are ultimately highly mismatched with the micelle‐like arrangement by means of components—domains with a structure that almost perfectly reproduces an arrangement with a centric hydrophobic core.

### Comparable analysis of human cystatin B with mutations introduced in positions 57 and 68

Mutant's structures of human cystatin B in the form of complex and single chains are available in PDB making possible the comparative analysis (Table 4) making the comparative analysis possible.

**Table 4:** List of human cystatin B mutants (listed in Table 2) available in PDB and their characteristics.

PDB – ID	Mutation	RD	*K*
3QRD – Dimer	L68V	0.677	1.0
Chain A		0.682	1.2
Chain B		0.697	1.3
A (10–59)		0.593	0.7
A (60–120)		0.559	0.7
B (10–59)		0.671	1.0
B (60–120)		0.541	0.6
A (10–59) + B (60–120)		0.560	0.6
B (10–59) + A (60–120)		0.507	0.5
3S67 – Chain	V57P	0.659	1.1
(10–59)		0.641	0.6
(60–120)		0.554	0.7
3SVA – Chain	V57D	0.673	1.2
(10–59)		0.607	0.6
(60–120)		0.571	0.7
3PS8 – Chain	L68V	0.661	1.1
(12–57)		0.540	0.5
(58–120)		0.557	0.7

**Fig. 12:**
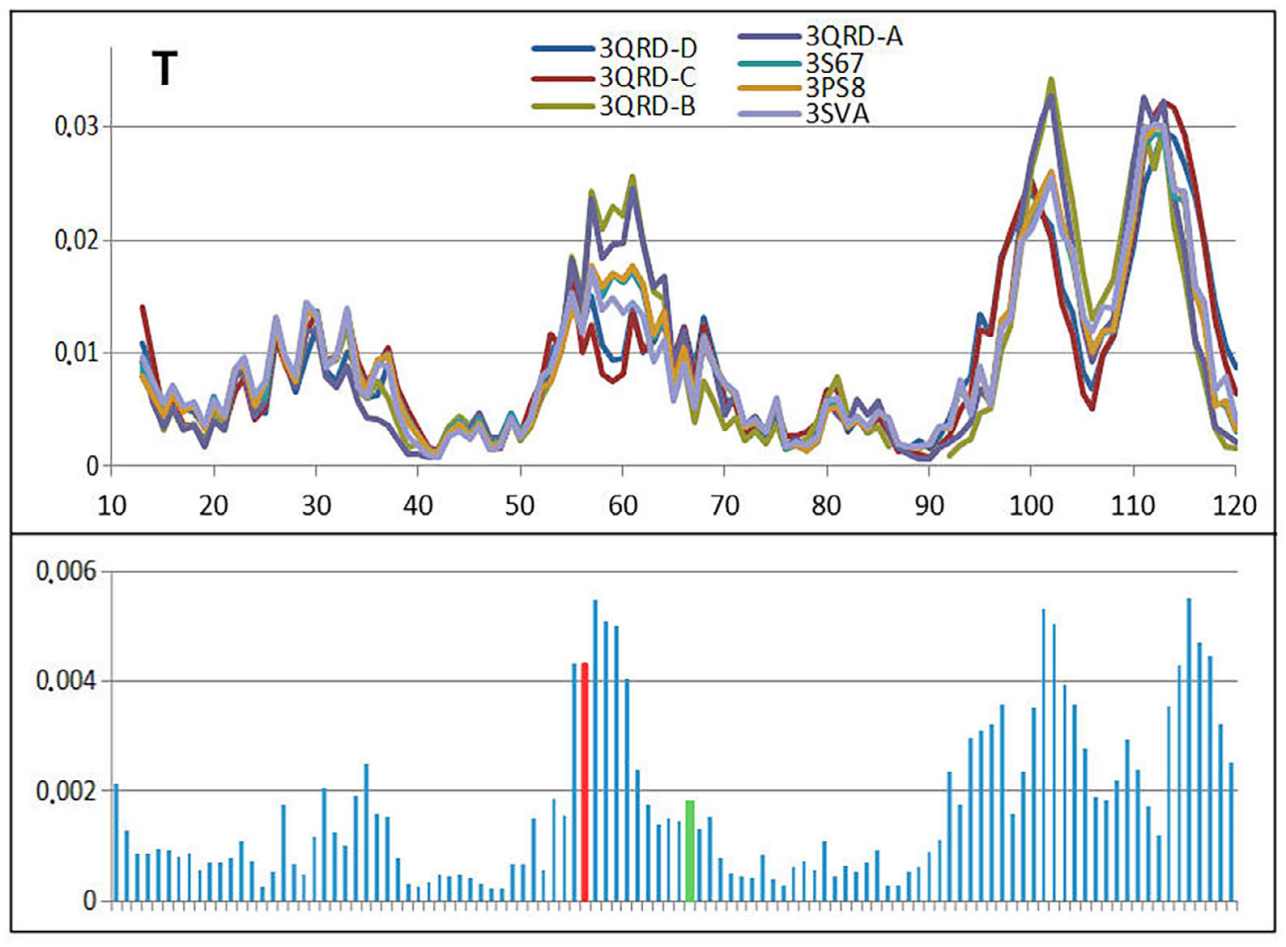
The set of *T* profiles for all discussed mutants revealing the differences between them together with standard deviation values for each position in the chain. The red and green bars distinguish positions of mutation.

**Fig. 13:**
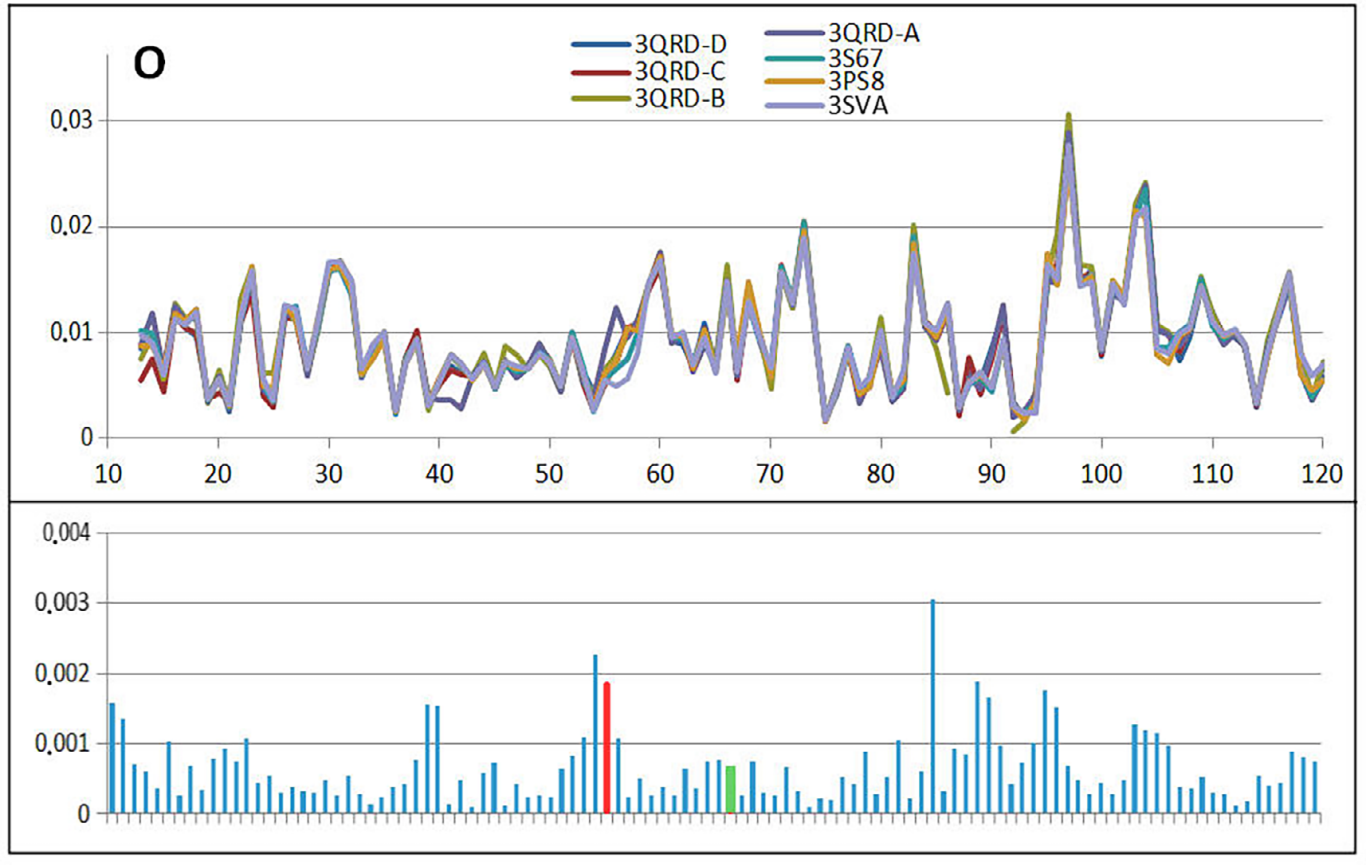
The set of *O* profiles for all discussed mutants revealing the differences between them together with standard deviation values for each position in the chain. The red and green bars distinguish positions of mutation.

**Fig. 14:**
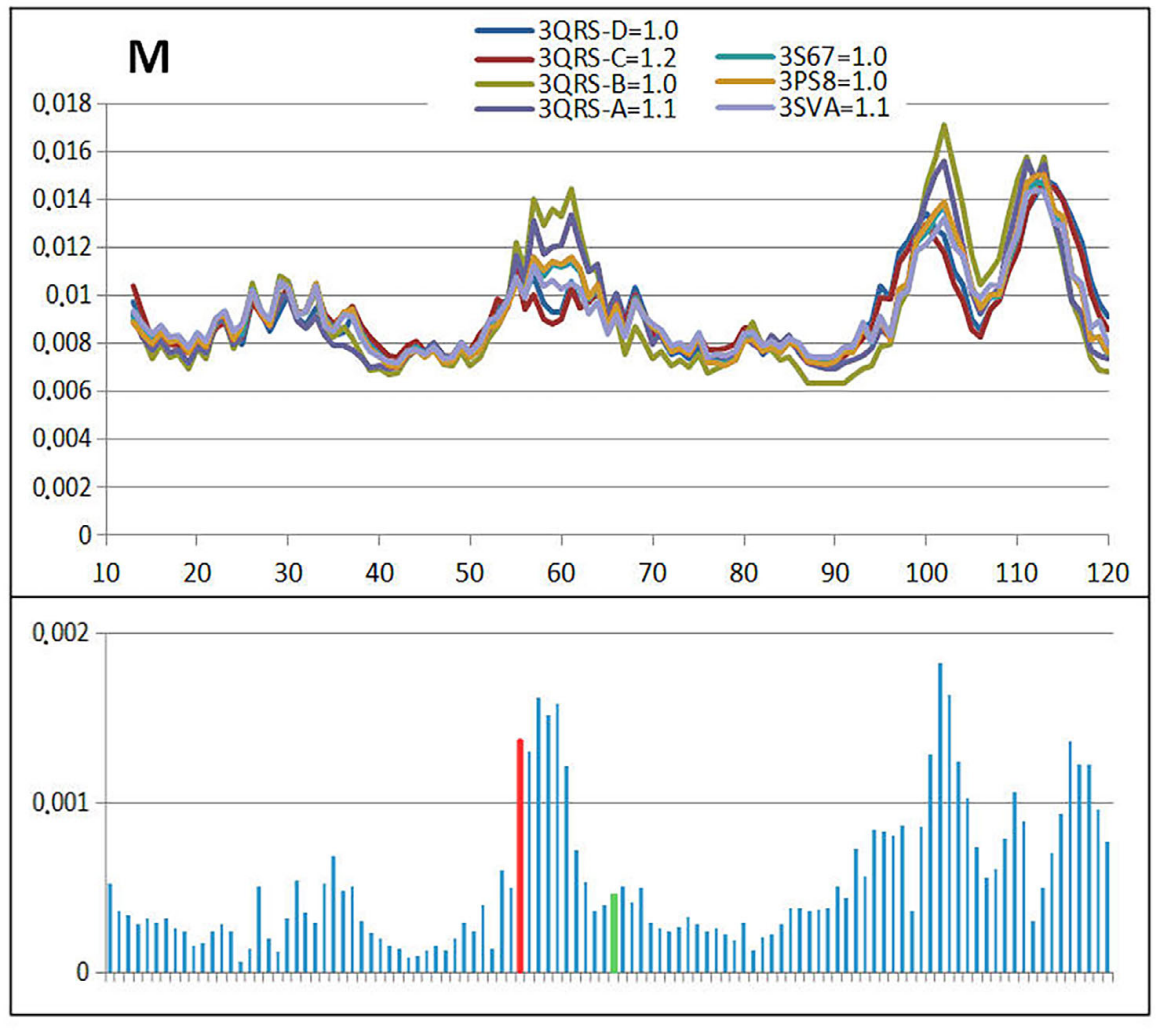
The set of M profiles for all discussed mutants revealing the differences between them together with standard deviation values for each position in the chain. The red and green bars distinguish positions of mutation.

**Fig. 15:**
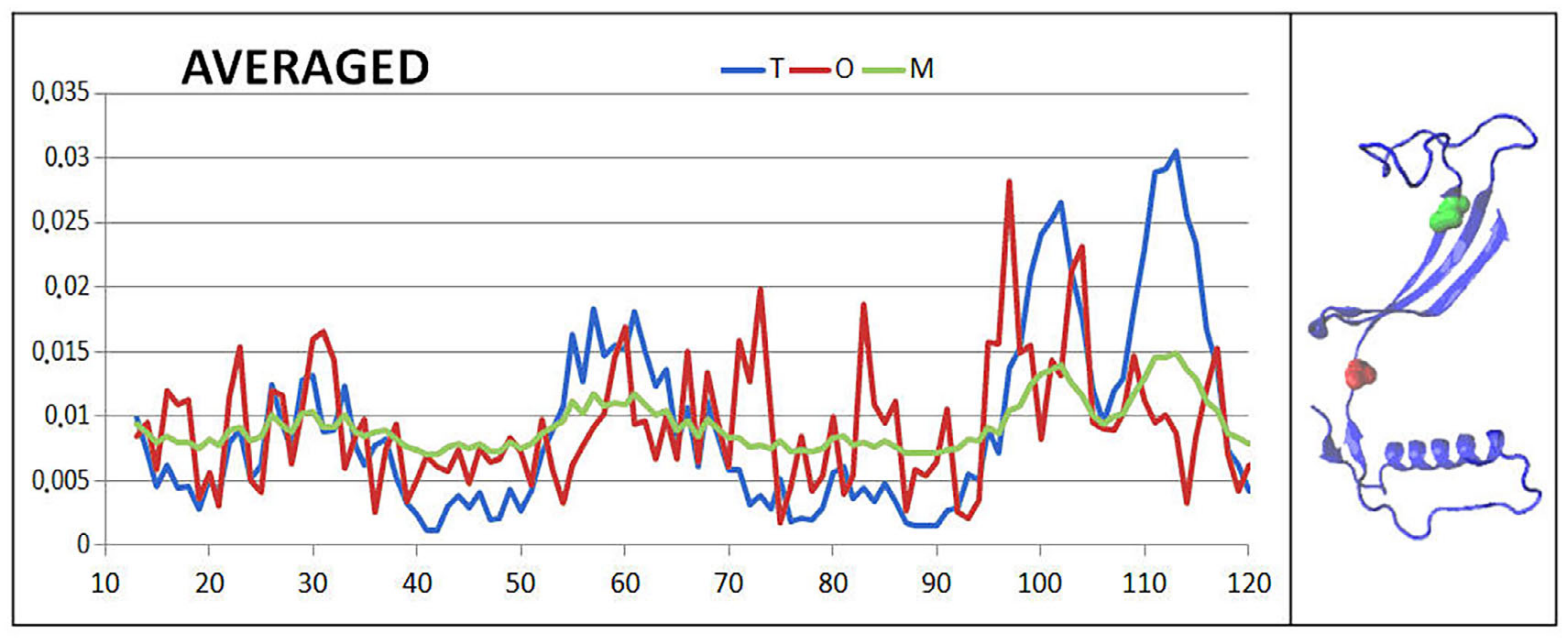
The *T*, *O* and M profiles representing the averaged *T*, *O* and M distributions together with the 3D presentation of discussed chain with mutated residues distinguished in red 57 and green 68.

Table 4 allows the comparable analysis of mutants present in human cystatin B revealing the similarity in respect to 2OTC presented in detail. The comparable analysis is shown presenting *T* (Fig. 12), *O* (Fig. 13) and M (Fig. 14) profiles for available structures of human cystatin B mutants. The significant differences in all presented profiles are observed close to mutated position 57, and however, the effects of mutations in position 68 is not that visible. The averaged profiles *T*, *O* and M with averaged *K* = 1.057 are shown in Fig. 15.

The highest differences in *T*_*i*_ hydrophobicity level are identified in chain fragment close to the position of mutation 57 (red bar on standard deviations) (Fig. 12). No such effect is observed for the mutation at position 68 (green bar on standard deviations). The mutation at position 57 (see Fig. 15) is in hinge region influencing relative position of the second domain (chain fragment following the mutation). This is why the maxima can be seen for standard deviation in C‐terminal domain. Thus, the values of *T*_*i*_ for this part of chain may differ.

The *O* profiles (Fig. 13) do not differ significantly due to identical sequence with only two positions mutated. The red and green bars in standard deviation positions are relatively low. The *O* distribution takes into account the inter‐residual interactions which in majority was similar. Substitution of Val by Leu or Val by Pro does not change much the intrinsical hydrophobicities (any scale of intrinsic hydrophobicity evaluates similar intrinsical hydrophobicity for these residues). Thus, the inter‐residual interaction is similar even for mutated residues.

The *M* profiles (Fig. 14) identify the mutation in position 57 as introducing significant differences in compared proteins. Similarly, *T* profiles (Fig. 12) mutation at the position 68 does not introduce significant differences in *M*_*i*_ values.

The set of averaged profiles *T*, *O* and *M* appears very similar in respect to 2OCT (Fig. 10). The only way to identify the differences is the presentation shown in Figs 12, 13, 14.

### Reference proteins of different status of domain swapping

Table 5 includes examples of proteins whose domains are built on the principle of domain swapping (there are examples with a structure consistent with the micelle‐like hydrophobicity system and those where this distribution is inconsistent with the 3D Gaussian distribution).

**Table 5:** List of reference proteins. The PDB – ID, activity, fragments participating in domain swapping and the values of the RD and *K* parameters are given. The proteins are divided according to the compatibility and incompatibility of the *T* distribution with respect to the *O* distribution. The text gives reasons for deviations from the proposed domain construction rule based on a common micelle‐like structure. Position with *—4 SS bonds and 2 interchains SS bonds; **—4SS bonds; ***—2 SS bonds.

PDB – ID	Name	Fragment		RD	*K*
		Chain A	Chain B		
**Micelle_like order**					
1N1C	Chaperone	20–130	131–195	0.446	0.4
3VM9	Myoglobin	1–81	82–153	0.410	0.3
1JWI	Toxin	(3–73) (96–125)	3–73	0.382	0.2
3NBS	Cytochrome	1–79	88–104	0.334	0.1
3S1U	Transferase	1–198	199–217	0.461	0.3
4AZM	Lipid binding	1–58	62–135	0.461	0.3
4P2X	Lyase	240–358	362–425	0.458	0.3
4ZCB	Lipid binding	1–55	59–133	0.450	0.3
5U6G	Transport	1–56	58–133	0.465	0.3
6J52	Apoptosis	1–50	50–94	0.330	0.0
6YRE	Transferase	1–196	199–217	0.475	0.4
**Order incompatible with micelle‐like**					
1BSR	Hydrolase	1–21	22–124	0.567	0.6
11BA*	Hydrolase	1–19	38–124	0.550	0.5
1JS0**	Hydrolase	1–113	114–124	0.558	0.6
1TIJ***	Inhibitor	9–58	59–120	0.563	0.5
4ESK	Immune system	26–33	35–120	0.585	0.6
6QKY	Lyase	28–58	61–257	0.520	0.5
6TP9	Cytochrome	4–31	29–88	0.725	1.2

Numerous domains are constructed on the principle of complementation, consistent with the hydrophobicity distribution model, by a fragment of another chain incorporated into the domain. In addition to such examples, examples with a distribution different from micelle‐like are also identified. This status may be the result of the presence of disulfide bonds, which impose structuring, often competing for the stabilisation of the tertiary structure with hydrophobic interactions including the hydrophobic core.

**Fig. 16:**
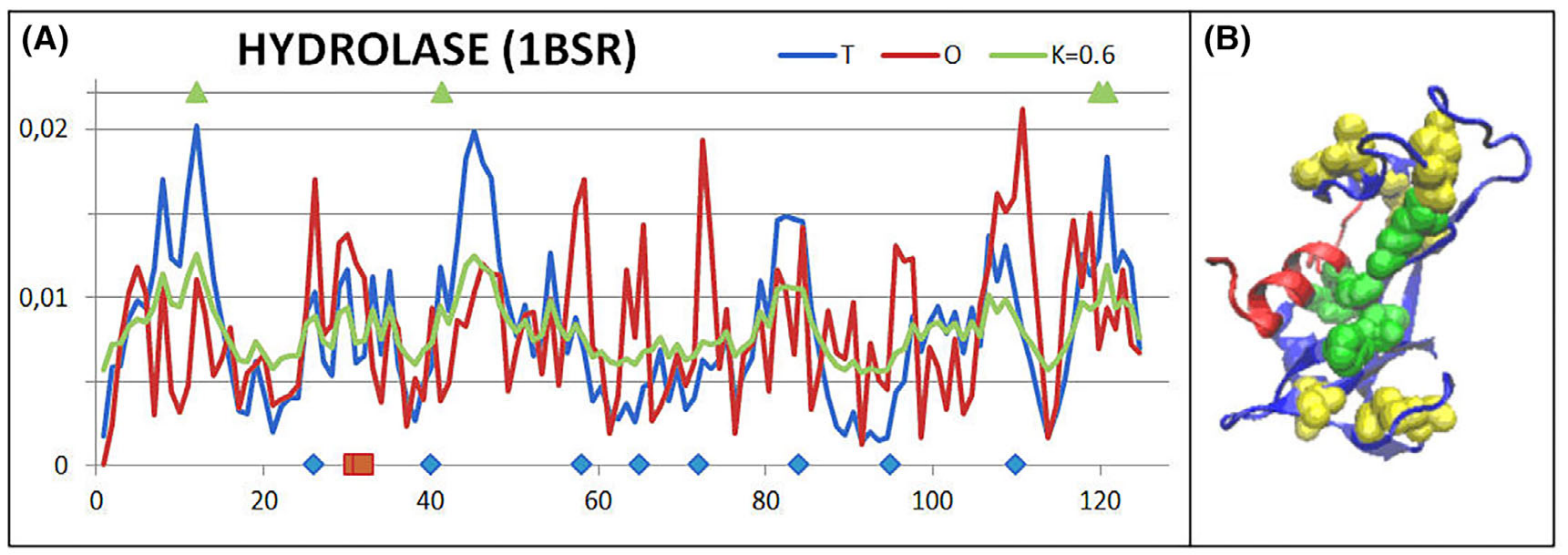
Hydrolase characterisation (1BSR). (A) Profiles *T* (blue), *O* (red) and *M* (green) for *K* = 0.6 describing the status of hydrolase domain (1BSR). Points on horizontal axis—top: green triangles—positions of catalytic residues; bottom—blue rhombus—positions of Cys in SS bonds, red squares—positions of Cys engaged in interchain SS bonds. (B) A 3D structure with highlighted positions of disulfide bonds (yellow) as well as the location of catalytic residues (green) is also shown. Red fragment – incorporated fragment from another chain.

The reasons for the high RD and *K* value for the domain with numerous disulfide bonds can be shown by the comparison of *T*, *O* and *M* profiles for RD = 0.567 and *K* = 0.6 that were identified in the hydrolase (1BSR [48]) (Fig. 16). The factor disturbing the compatibility of the *T* and *O* distribution are the Cys positions that constitute SS bonds. They show increased levels of *O*_*i*_ due to their close proximity and high intrinsic hydrophobicity value.

Additionally, cysteines involved in interchain bonds are located nearby, which also causes a significant increase in the *O*_*i*_ value. The differentiation of *T*_*i*_ and *O*_*i*_ levels is also influenced by catalytic residues, which are located in the cavity prepared for interaction with the substrate. In the case of the domain discussed here, any inconsistencies in the *T* and *O* distributions result from adaptation to the biological function performed. This state of affairs is typical and characteristic of proteins with a specific biological function, where the specificity requires local incompatibility of *T* and *O* distributions in order to write the appropriate structural code guaranteeing biological activity [12]. Figure 16B shows the substrate binding cavity, which is expressed by a local hydrophobicity deficit.

The analysis of the influence and relationship between these two stabilisation factors discussed in [49] reveals a common pursuit of stabilisation (SS bonds present and hydrophobic core present). The superiority of the disulfide bond system is also visible, causing a significant disruption of the structure of the hydrophobic core, including its complete deprivation. Examples of such structures are as follows: 3QRD [39], 1TIJ [35], 1JS0 [34], 1BSR [48] (Table 5).

Another example with RD > 0.5 is domains in which the active centre associated with biological activity is located. The domains of immunoglobulins carrying a tool for recognising and complexing another protein show local distributions inconsistent with the micelle‐like system. Similarly, enzymes—including lyases (6QKY)—represent hydrophobicity distributions in the far form towards micelle‐like. A detailed analysis of representatives of enzyme classes [44] shows just such a status. It is therefore not surprising that the domain of this enzyme's molecule has encoded local inconsistencies with the micelle‐like distribution in its structure related to its function.

An extremely inconsistent *O* distribution with the *T* distribution was identified in the protein responsible for electron transfer (6TP9). It should be noted that the structure available in PDB is in the form of a complex consisting of many chains. The presence of these numerous chains may act as an external force field for the analysed domain. This example may be very specific in the context of other proteins analysed here, hence the unusual characteristics of this protein, especially since there is another cytochrome on the list (Table 5) whose domain shows perfect adaptation to the micelle‐like distribution of hydrophobicity.

## Discussion

The structural arrangement presented here, constructed based on domain swapping, appears to be a way to obtain a stable structure with encoded limited flexibility within the dimers discussed. It is assumed that single chains constitute a sort of ‘permanent chaperone’ arrangement in the domain‐swapping system guaranteeing that a structure with ensured biological activity is maintained. In the case of SARS‐CoV main protease, the status of the catalytic residues matches the micelle‐like arrangement (Fig. 7A,C), representing levels of hydrophobicity corresponding to expectations, consistent with the micelle‐like arrangement. These domains can therefore be formed in an aqueous environment by a spontaneous process directed by an external force field in the form of polar water. The reciprocal arrangement of the domains, on the other hand, dictated by the domain‐swapping arrangement, leads to their localisation showing a record of specificity. This is because these residues within the dimer exhibit a local excess of hydrophobicity (Fig. 6). Excess hydrophobicity can result in the complexation of another protein with a comparable level of surface exposure. However, this is not the case here. Completely different chain fragments, including those involved in domain swapping, ensure that the catalytic residues are unavailable to the other protein. Dom3 (Fig. 6) and the stabilising domain‐swapping system are responsible for constructing the interface.

The model applied for analysis of domain‐swapping structures appears to be able to identify the structural consequences of introduced mutations. It was shown using the mutants of human cystatin B.

## Conclusions

The construction mechanism for domains formed by the exchange of polypeptide chain sections (domain swapping) is based, in the proteins discussed, on the contribution of the inserted section to the construction of the common hydrophobic core. The question relates to the need to swap chain sections in place of inserting one's own chain section. This appears to be associated with the encoded form of domain mobility when maintaining a two‐chain structure. The final native structural form is constructed from components (domains) with high stability resulting from an ordering with a centric hydrophobic core. The contribution of the aforementioned sections is related to the specific form of stabilisation of the entire structure. This is why this problem is also discussed in the context of misfolding [50, 51, 52, 53]. Individual domains exhibiting high stability combined in a way resulting in an arrangement with reduced stability turn out to be a way to achieve adequate mobility of individual domains while maintaining their internal stability. In addition, the flexibility/stability relationship appears to be positioned throughout the structure. Furthermore, the acquisition of stable domains in the case of domain swapping is achieved in an aqueous environment in a way that does not require the contribution of environmental factors other than water.

## Conflict of interest

The authors declare no conflict of interest.

### Peer review

The peer review history for this article is available at https://www.webofscience.com/api/gateway/wos/peer‐review/10.1002/2211‐5463.13911.

## Author contributions

IR and LK contributed to conceptualization. KS contributed to methodology, validation. KS and DD contributed to software, data curation. IR and LK contributed to formal analysis. IR contributed to investigation, supervision, project administration, funding acquisition and resources. KS and IR contributed to writing—original draft preparation; writing—review, editing and visualisation. All authors have read and agreed to the published version of the manuscript.

## Data Availability

All data can be available on request addressed to the corresponding author. The program allowing calculation of RD is accessible on GitHub platform: https://github.com/KatarzynaStapor/FODmodel and on the platform https://hphob.sano.science.
